# Ischemic stroke risk, smoking, and the genetics of inflammation in a biracial population: the stroke prevention in young women study

**DOI:** 10.1186/1477-9560-6-11

**Published:** 2008-08-26

**Authors:** John W Cole, David W Brown, Wayne H Giles, Oscar C Stine, Jeffrey R O'Connell, Braxton D Mitchell, John D Sorkin, Marcella A Wozniak, Barney J Stern, Mary J Sparks, Mark T Dobbins, Latasha T Shoffner, Nancy K Zappala, Laurie J Reinhart, Steven J Kittner

**Affiliations:** 1 Veterans Affairs Medical Center, Baltimore, Maryland, USA; 2 Department of Neurology, University of Maryland School of Medicine, Baltimore, Maryland, USA; 3 Centers for Disease Control, Atlanta, Georgia, USA; 4 Department of Epidemiology and Preventive Medicine, University of Maryland School of Medicine, Baltimore, Maryland, USA; 5 Department of Medicine, University of Maryland School of Medicine, Baltimore, Maryland, USA; 6 University of Maryland School of Medicine Claude D. Pepper Older Americans Independence Center, Maryland, USA

## Abstract

**Background:**

Although cigarette smoking is a well-established risk factor for vascular disease, the genetic mechanisms that link cigarette smoking to an increased incidence of stroke are not well understood. Genetic variations within the genes of the inflammatory pathways are thought to partially mediate this risk. Here we evaluate the association of several inflammatory gene single nucleotide polymorphisms (SNPs) with ischemic stroke risk among young women, further stratified by current cigarette smoking status.

**Methods:**

A population-based case-control study of stroke among women aged 15–49 identified 224 cases of first ischemic stroke (47.3% African-American) and 211 age-comparable control subjects (43.1% African-American). Several inflammatory candidate gene SNPs chosen through literature review were genotyped in the study population and assessed for association with stroke and interaction with smoking status.

**Results:**

Of the 8 SNPs (across 6 genes) analyzed, only *IL6 *SNP rs2069832 (allele C, African-American frequency = 92%, Caucasian frequency = 55%) was found to be significantly associated with stroke using an additive model, and this was only among African-Americans (age-adjusted: OR = 2.2, 95% CI = 1.0–5.0, p = 0.049; risk factor adjusted: OR = 2.5, 95% CI = 1.0–6.5, p = 0.05). When stratified by smoking status, two SNPs demonstrated statistically significant gene-environment interactions. First, the T allele (frequency = 5%) of *IL6 *SNP rs2069830 was found to be protective among non-smokers (OR = 0.30, 95% CI = 0.11–.082, p = 0.02), but not among smokers (OR = 1.63, 95% CI = 0.48–5.58, p = 0.43); genotype by smoking interaction (p = 0.036). Second, the C allele (frequency = 39%) of *CD14 *SNP rs2569190 was found to increase risk among smokers (OR = 2.05, 95% CI = 1.09–3.86, p = 0.03), but not among non-smokers (OR = 0.93, 95% CI = 0.62–1.39, p = 0.72); genotype by smoking interaction (p = 0.039).

**Conclusion:**

This study demonstrates that inflammatory gene SNPs are associated with early-onset ischemic stroke among African-American women (*IL6*) and that cigarette smoking may modulate stroke risk through a gene-environment interaction (*IL6 and CD14*). Our finding replicates a prior study showing an interaction with smoking and the C allele of *CD14 *SNP rs2569190.

## Introduction

Cigarette smoking is a well-established risk factor for vascular disease and stroke. Various mechanisms that link cigarette smoking to these risks have been described, including: vasomotor dysfunction [1-3] modification of the lipid profile (notably increased oxidation of LDL) [1,2,4], modification of prothrombotic effects, including altered platelet function [2,5], and deregulation of antithrombotic, prothrombotic, and fibrinolytic mechanisms [1,2]. The final common pathway shared by these mechanisms is heightened inflammation, which is also considered a potential risk mechanism [1,2,6]. Each of the mechanisms is under the influence of multiple genes, therefore an individuals' susceptibility to the detrimental effects of cigarette smoke appears to be related to their pro-inflammatory versus anti-inflammatory gene-specific allele load. This hypothesis is supported by multiple studies that indicate pro-inflammatory gene-specific allele load is associated with enhanced inflammation and early atherosclerosis in smokers [7-10]. Many inflammatory genes have been implicated in these relationships, specific to this study these include: interleukin-1A (*IL1A; *OMIM 147760), interleukin-1 receptor – type I precursor (*IL1R1; *OMIM 147810), interleukin-1B (*IL1B; *OMIM 147720), interleukin-6 (*IL6; *OMIM 147620), stromelysin-1 (*MMP3; *OMIM 185250), and monocyte endotoxin receptor (*CD14; *OMIM 158120). In this study, we analyze the relationships between various polymorphisms of these genes, ischemic stroke risk, and smoking status.

## Research design and methods

### Study subjects

The Stroke Prevention in Young Women Study 2 (SPYW2) is a population-based case-control study that was designed to examine genetic risk factors for ischemic stroke in young women. The term "population-based" indicates that the cases and the comparison control group were identified from the same population including all of Maryland (except the far Western panhandle), Washington DC, and the southern portions of both Pennsylvania and Delaware. Two hundred thirty nine female cases age 15 to 49 years of age with a first cerebral infarction were identified by discharge surveillance at 51 regional hospitals and through direct referral by regional neurologists. The methods for discharge surveillance, chart abstraction, and case adjudication have been described previously [11-13]. We determined each subject's case-control status (i.e. determined subjects who had a stroke) blinded to genetic information. Strokes were classified as having a probable, possible or undetermined etiology [11,12]. Using predetermined exclusion criteria, modified from the Siblings With Ischemic Stroke Study (SWISS) protocol [14], we excluded 15 cases with the following characteristics: sickle cell disease (n = 1), CNS vasculitis by angiogram and clinical criteria (n = 3), post-radiation arteriopathy (n = 1), endocarditis (n = 3), neurosyphillis (n = 1), mechanical prosthetic heart valves (n = 2), left atrial myxoma (n = 1), and cocaine use in the 48 hours prior to their stroke (n = 3). Controls subjects (212 women without a history of stroke), were identified by random digit dialing and were frequency matched to the cases by age and geographic region of residence. One control was excluded from analyses based on a history of sickle cell disease. Thus, the sample for genetic analyses consisted of 224 cases and 211 controls.

Cases and controls were grouped into the following race-ethnic categories: Caucasian (non-Hispanic) (95 cases and 99 controls), African-American (105 cases and 91 controls), and other (including Hispanic, Asian, American-Indian, etc.) (24 cases and 21 controls). Because of the small size and heterogeneity of the latter group, it was not analyzed separately, but was included within the combined total study group (224 cases and 211 controls). Strokes were further classified by subtype: the atherosclerotic group included 27 cases with either probable or possible atherosclerotic mechanism, the cardiac group included 14 cases with a probable cardiac source of embolism, the probable dissection group included 13 cases confirmed by neuroimaging, the lacunar group included 45 cases of symptomatic small deep lesions on neuroimaging studies or classic lacunar syndromes regardless of other potential causes, and the probable hematologic group included 9 cases. These categories were not mutually exclusive. There were 125 non-lacunar stroke cases of undetermined etiology.

### Single Nucleotide Polymorphisms (SNPs) evaluated

We identified genes and SNPs that influence inflammation through a literature review seeking an association of the genes and SNPs with vascular disease (stroke, myocardial infarction, peripheral vascular disease, or atherosclerosis). If the evidence suggested that smoking might modify the relationship between gene or SNP and stroke through a gene-environment interaction, a higher priority for inclusion in our analyses was assigned to that gene or SNP. Because we sought to evaluate common SNPs, only those SNPs with a minor allele frequency of 0.05 or greater among African-Americans or Caucasians as per the NCBI SNP website  were evaluated. After weighing these considerations, the final list of genes and SNPs analyzed in this study included: *CD14 *(rs2569190), *IL1A *(rs17561), *IL1R1 *(rs3917318), *IL1B *(rs3917365), *IL6 *(rs1800797, rs2069830, 2069832), and *MMP3 *(rs679620).

### Genotyping methods for the case/control population

Genotyping was conducted on DNA isolated from whole blood using the QIAamp DNA Blood Maxi Kit (Qiagen, Valencia, CA). SNP genotyping was performed by one of two methods. The first method, developed for a SNP stream Ultra-High Throughput machine (Beckman Coulter, Inc., Fullerton, CA) is capable of genotyping up to 12-SNPs simultaneously. Sequences surrounding the SNPs were obtained from GenBank  and submitted to Autoprimer.com (Beckman Coulter, Inc.). For each SNP, three primers were designed, two for PCR amplification and an internal primer with a 5' DNA sequence tag. Pairs of primers were used to initiate PCR amplification. The free primers were removed by enzymatic digestion using Exonuclease I and Shrimp Alkaline Phosphotase (Beckman Coulter, Inc.). The internal primers were used to initiate a sequencing reaction that adds one labeled base for the alternative nucleotides of each SNP to have distinct labels. The labeled products are separated on a SNP-IT plate consisting of 384 mini-arrays with 16-spots each (Beckman Coulter, Inc.). For each individual DNA sample, 16 spots hybridized to the two homozygotes, the heterozygote, a negative control and the 12-labeled primers associated with the 12-SNPs. Thus, every PCR and labeling reaction had internal controls to confirm the success of the reactions and the appropriateness of the fluorescent outputs for each DNA sample. SNPs genotyped using this method include: *IL1R1 *(rs3917318); *IL1B *(rs3917365); *IL6 *(rs1800797, 2069832).

The second genotyping method was Taqman (Applied Biosystems). This method is based on four primers, two flanking the SNP that are used to amplify the DNA surrounding the SNP and two, one for each alternative allele, that were labeled with different fluorescent dyes. The original form of the labeled primer has a quencher in close proximity to the dye. However, when the exonuclease activity of DNA polymerase disrupts the primer hybridized to the single strand DNA during the PCR, then quencher and the dye are released and the fluorescence can be measured. The reaction itself follows manufacturer's instructions included with each individual primer set. SNPs genotyped using this method include: *CD14 *(rs2569190); *IL1A *(rs17561); *IL6 *(rs2069830); *MMP3 *(rs679620).

### Analyses

All statistical analyses were performed using SAS^®^, Version 9.1 (SAS Institute, Cary, NC). We compared means by two-sided t-tests and proportions by χ^2 ^tests. All SNPs were verified to be in Hardy-Weinberg equilibrium. Adjusted odds ratios from logistic regression were used to determine whether the presence of the risk allele was associated with an increased risk for ischemic stroke after controlling for potential confounders.

Two additive models were used to determine the relation between SNP and outcome. The first model (our primary analyses) was adjusted for age and race (when all subjects were included in the analysis) or only age when the analysis was stratified by race (i.e. African-Americans and Caucasians). The second model, (adjusted model) added history of hypertension, diabetes, smoking, oral contraceptive (OCP) use and angina pectoris or myocardial infarction (angina-MI), to age (or age and race) to the independent variables used in the first model. The results of the analyses were expressed as odds ratios which quantify the increased risk of ischemic stroke associated with each additional risk allele. Age, race, current cigarette smoking status, and OCP use were determined by subject reports (or proxy report, if a participant was unable to answer). Hypertension and diabetes mellitus were determined by asking study participants (or a proxy) if a physician had ever told them they had the condition. Current smoking was defined as having one or more cigarettes in the month (31 days) preceding their stroke, or for controls, in the month prior to their interview. Current oral contraceptive use was also defined as use in the prior month.

In order to evaluate each SNP for a genotype by smoking interaction (as per our initial hypothesis), study participants were stratified by smoking status and evaluated in an age-, race-adjusted additive model. For those SNPs that demonstrated a potential genotype by smoking interaction the significance of the difference between the point estimates (i.e. the log odds ratios) in smokers and non-smokers was tested using a z-test. Lastly, SNPs with significant z-test results were additionally evaluated for potential race-specific smoking interactions in an age-adjusted additive model, with the SNP stratified by smoking status further stratified by race (Caucasian and African-American).

In secondary analyses, significantly associated SNPs from the primary analyses (total population and race-stratified) underwent further analyses to evaluate groups stratified by other standard risk factors (age, hypertension, diabetes mellitus, OCP use, and history of angina/MI) and ischemic stroke subtype (atherosclerotic, cardiac, dissection, lacunar, hematologic, and stroke of unknown etiology (i.e. all other stroke)) with the later groups compared to all controls. Additionally, analyses consisting of dominant and recessive genetic models that included similar groups and covariates to the primary analyses were also performed. We did not adjust for multiple comparisons because our study was considered to be hypothesis generating.

## Results

### Subject characteristics

Demographic and risk factor characteristics by case-control status are described in Table 1. The mean age of the cases was 41.7 years and the mean age of control subjects was 39.6 years. Cases were significantly more likely than controls to have a history of hypertension (p < 0.0001), diabetes (p = 0.0002), angina-MI (p = 0.0005), to currently smoke cigarettes (p < 0.0001), and to report the use of oral contraceptive pills (OCP) within the month prior to their stroke (p = 0.032).

**Table 1:** Characteristics, by case-control status.

	**Cases (N = 224)**	**Controls (N = 211)**	**p-value**
Mean age (years)	41.7	39.6	0.0026
African-American (%)	47.3	43.1	0.579
Hypertension (%)	41.1	14.2	<.0001
Diabetes mellitus (%)	17.9	6.2	0.0002
Current smokers (%)	47.8	23.7	<.0001
Angina-MI (%)	11.6	2.8	0.0005
OCP (%)*	12.2	6.2	0.032

* Two cases and one control could not recall their last OCP use, therefore cases N = 222 and controls N = 210.

### Ischemic stroke risk

Table 2 gives p-values for the age-adjusted additive model and risk factor-adjusted additive model for the eight SNPs stratified by race and case-control status, and also provides each SNPs location, allelic variants, and genotype call rate. Of the SNPs analyzed, only the *IL6 *SNP rs2069832 (C allele; overall frequency: African-Americans = 92%, Caucasians = 55%) was found to be significantly associated with stroke, and this was only among African-Americans (Age-adjusted model: OR = 2.2, 95% CI = 1.0–5.0, p = 0.049; Risk Factor model: OR = 2.5, 95% CI = 1.0–6.5, p = 0.05). Dominant and recessive models did not strengthen the association (data not shown). Stratifying participants by other risk factors and evaluating stroke risk in relation to SNP rs2069832 genotype revealed no associations with stroke (data not shown). Stratifying participants by stroke subtype as compared to all controls and evaluating stroke risk in relation to SNP rs2069832 genotype revealed no significant associations (data not shown).

**Table 2:** Proinflammatory SNPs stratified by race and case/control status demonstrating allelic variants, genotype call rate, minor allele frequencies and p-values for age-adjusted additive model and risk factor adjusted additive model.

**Gene**	**SNP rs number, location and function**	Alleles*	**Call Rate (%)**	**African-Americans**				**Caucasians**			
				**Cases**	**Controls**	**p-value**		**Cases**	**Controls**	**p-value**	

				**Minor Allele Frequency/N**	**Minor Allele Frequency/N**	**Age-adjusted**	Risk factor adjusted**	**Minor Allele Frequency/N**	**Minor Allele Frequency/N**	**Age-adjusted**	Risk factor adjusted**

*CD14*^ψ^	rs2569190, 5' promotor	C/**T**	99%	0.38/77	0.42/62	0.57	0.66	0.49/65	0.40/75	0.13	0.30
*IL1A*	rs17561, exon 4 nonsynonymous	G/**T**	98%	0.18/102	0.18/90	0.83	0.73	0.27/93	0.31/94	0.32	0.80
*IL1R1*	rs3917318, intron 10	A/**G**	91%	0.33/97	0.29/88	0.44	0.23	0.33/92	0.36/92	0.53	0.24
ILIB	rs3917365, 3' near gene	C/**T**	81%	0.21/97	0.17/83	0.34	0.36	0.08/86	0.08/89	0.85	0.87
*IL6*	rs1800797, 5' near gene	**A**/G	91%	0.06/99	0.10/88	0.19	0.18	0.42/93	0.43/91	0.89	0.87
	rs2069830, exon 2 nonsynonymous	C/**T**	91%	0.07/100	0.09/87	0.32	0.31	0.01/90	0.03/93	0.30	0.35
	rs2069832, intron 2	C/**T**	89%	0.05/97	0.12/86	**0.049**	**0.05**	0.45/92	0.45/91	0.97	0.85
*MMP3*	rs679620, exon 2 nonsynonymous	C/**T**	89%	0.35/103	0.35/88	0.95	0.72	0.45/90	0.47/96	0.85	1.0

^ψ^*CD14 *SNP rs2569190 was genotyped in a smaller case-control sample (N = 315).

* Minor allele in African-Americans (bolded in column 3 above).

** Risk factor adjusted – Additive model adjusted for age, hypertension, diabetes, oral contraceptive use, and angina-MI.

### Ischemic stroke risk as stratified by smoking status

Table 3 demonstrates the age- and race-adjusted additive model results for the SNPs stratified by smoking status. Two SNPs demonstrated a gene-environment interaction based upon smoking status. First, *CD-14 *SNP rs2569190 (C allele; frequency in total population = 39%) was found to be associated with increased risk of stroke among smokers (OR = 2.05, 95% CI = 1.09–3.87, p = 0.027), but not non-smokers (OR = 0.93, 95% CI = 0.62–1.39, p = 0.72). The two odds ratios were significantly different (p = 0.039). Secondly, *IL6 *SNP rs2069830 (T allele; frequency in total population = 5%) was found to be protective among non-smokers (OR = 0.30, 95% CI = 0.11–.082, p = 0.02), but not among smokers (OR = 1.63, 95% CI = 0.48–5.58, p = 0.43). The two odds ratios were significantly different (p = 0.036).

**Table 3:** Proinflammatory SNPs stratified by smoking status demonstrating the number of cases and controls, and Odds Ratio, 95%CI and p-values in an age-, race-adjusted additive model.

**Gene**	**rs number**	**Smokers**				**Non-Smokers**			
		**Cases/Controls**	OR*	**95% CI**	**p-value**	**Cases/Controls**	OR*	**95% CI**	**p-value**

*CD14*	rs2569190	77/31	**2.05**	**1.09–3.87**	**0.027**	82/121	0.93	0.62–1.39	0.72
*IL1A*	rs17561	106/48	1.68	0.84–3.32	0.14	111/156	1.05	0.69–1.60	0.83
*IL1R1*	rs3917318	102/45	1.138	0.67–1.94	0.64	108/155	1.07	0.74–1.54	0.72
IL1B	rs3917365	99/44	1.63	0.78–3.42	0.19	105/146	1.03	0.62–1.73	0.90
*IL6*	rs1800797	104/43	1.28	0.66–2.46	0.46	110/155	1.05	0.67–1.64	0.83
	rs2069830	103/45	1.63	0.48–5.58	0.43	109/155	**0.30**	**0.11–0.82**	**0.019**
	rs2069832	101/45	0.69	0.35–1.35	0.28	109/152	0.80	0.51–1.26	0.34
*MMP3*	rs679620	103/46	0.98	0.58–1.65	0.93	110/157	1.01	0.71–1.43	0.97

* Adjusted for age and race (African-American, Caucasian, and other).

### Ischemic stroke risk as stratified by smoking status further stratified by race

Results of the race-stratified smoking-genotype interaction analyses demonstrated no significant interactions. However, the race-stratified point estimates (ORs) remained similar to the combined population point estimates as detailed in the preceding paragraph. For CD-14 SNP rs2569190, similar ORs were seen among both smokers of both races (Caucasian OR = 2.21, p = 0.127; African-American OR = 1.91, p = 0.143) and non-smokers of both races (Caucasian OR = 1.18, p = 0.60; African-American OR = 0.83, p = 0.55). For IL-6 SNP rs2069830, among African-Americans, similar ORs as found in the combined population were seen among smokers (OR = 1.31, p = 0.71) and non-smokers (OR = 0.38, p = 0.10), consistent with a protective effect for the T allele among non-smokers. For IL-6 SNP rs2069830, among both Caucasian non-smokers and smokers, the logistic regression models did not converge due to the low frequency (2%) of the T allele.

## Discussion

Our study did not demonstrate strong relationships between the inflammatory gene polymorphisms and ischemic stroke risk. Only *IL6 *(rs2069832) was found to be associated with stroke, and only among African-Americans. However, it did identify two polymorphisms that appear to influence stroke risk via a gene-environment interaction with cigarette smoking, IL6 rs2069830 and CD-14 rs2569190.

IL-6 plays a key role in the acute inflammatory response and in regulation of the production of acute phase proteins such as C-reactive protein [15,16]. It contributes to the inflammatory response by activating endothelial cells [15,17] and stimulating the synthesis of fibrinogen [15,18]. In vivo exposure to cigarette smoke has been shown to increase the expression of proinflammatory cytokines including IL-6 [19]. Furthermore, several studies have demonstrated that IL-6 polymorphisms may mediate carotid artery intima-media wall thickness [20,21], an intermediate marker of stroke risk. Our results, consistent with other studies, demonstrates that genetic variation in IL-6 may modify stroke risk [15,22], and that this increased risk may be due to synergistic effects between proinflammatory genotypes and smoking [23].

CD14 is a surface protein preferentially expressed on monocytes and macrophages that acts to bind lipopolysaccharide-binding protein (also called endotoxin). Endotoxin is a potent mediator of inflammation, and smokers have elevated plasma levels of endotoxin. Hence, it is believed that the risk of atherosclerosis from endotoxemia is increased in smokers [24]. The *CD14 SNP *rs2569190 is a C > T substitution in the proximal *CD14 *promoter GC box at position -260 from the translation start site and results in a *Hae*III restriction site [25]. The results of studies which have tried to determine which allele mediates vascular risk have been variable. The T allele was associated with an increased risk of MI [25-28] and two ischemic stroke subtypes including atherosclerosis of large arteries and microangiopathy [29]. Contrary to these studies, the C allele was associated with a higher risk of carotid plaque formation [30], and was found to act synergistically with other inflammatory SNPs to increase carotid IMT [7,9], specifically among smokers [7]. Still other studies evaluating this SNP have found no association with stroke [31,32]. Our results support a gene-environment association between the C allele and ischemic stroke risk among smokers. Our findings replicate a prior study showing an interaction with smoking and the C allele of *CD14 *SNP rs2569190 [7].

Our study has several limitations. Most notably, we only evaluated a limited number of SNPs, which were not sufficient to comprehensively evaluate stroke risk or smoking interaction for any of the genes studied. Secondly, our study did not provide any mechanistic information regarding stroke risk or protection. Specifically, we did not determine if our SNPs were associated with increased or decreased protein activity. Furthermore, our study population is relatively small and is underpowered to detect modest effects, particularly in the stroke subtype analyses. Although we have attempted to minimize phenotypic heterogeneity through identifying ischemic stroke subtypes during adjudication, we recognize that residual phenotypic heterogeneity may exist. Lastly, though we performed numerous analyses, no correction was made for multiple comparisons. This allows for the possibility that our results could be attained through chance alone, although confirming CD-14 rs2569190 as a mediator of vascular risk, and specifically among smokers of both races, makes such a false positive association less likely.

## Conclusion

This study demonstrates that inflammatory gene SNPs may be associated with early-onset ischemic stroke among African-American women (*IL6*) and that cigarette smoking may modulate stroke risk through a gene-environment interaction (*IL6 and CD14*).

## Competing interests

The authors declare that they have no competing interests.

## Authors' contributions

All authors certify that they participated in the conceptual design of this work, the analysis of the data, and the writing of the manuscript to take public responsibility for it. All authors reviewed the final version of the manuscript and approve it for publication. JWC, OCS, and SJK participated in the writing of the initial draft. OCS participated in the genotyping. JWC OCS, MAW, BJS, MJS, MD, LS, NZ, and SJK participated in DNA and data collection. JWC, DWB, WHG, JRO, BDM, JDS, and LJR participated in the data analysis. All authors provided critiques of the final manuscript.

The findings and conclusions in this report are those of the authors and do not necessarily represent the official position of the Centers for Disease Control and Prevention.

## Availability & requirements




